# CT-Based Prediction of Liver Function and Post-PVE Hypertrophy Using an Artificial Neural Network

**DOI:** 10.3390/jcm10143079

**Published:** 2021-07-12

**Authors:** Daniel Heise, Maximilian Schulze-Hagen, Jan Bednarsch, Roman Eickhoff, Andreas Kroh, Philipp Bruners, Simon B. Eickhoff, Ralph Brecheisen, Florian Ulmer, Ulf Peter Neumann

**Affiliations:** 1 Department of Surgery and Transplantation, University Hospital RWTH Aachen, 52074 Aachen, Germany; jbednarsch@ukaachen.de (J.B.); reickhoff@ukaachen.de (R.E.); akroh@ukaachen.de (A.K.); fulmer@ukaachen.de (F.U.); uneumann@ukaachen.de (U.P.N.); 2 Department of Diagnostic and Interventional Radiology, RWTH Aachen University Hospital, 52074 Aachen, Germany; mschulze@ukaachen.de (M.S.-H.); pbruners@ukaachen.de (P.B.); 3 Research Center Juelich, Institute of Neuroscience and Medicine, Brain & Behaviour (INM-7), 52074 Juelich, Germany; simon.b.eickhoff@gmail.com; 4 Institute of Systems Neuroscience, Medical Faculty, Heinrich Heine University Duesseldorf, 40225 Duesseldorf, Germany; 5 Department of Surgery, Maastricht University Medical Centre (MUMC), 6229 Maastricht, The Netherlands; r.brecheisen@maastrichtuniversity.nl

**Keywords:** liver function, liver volume, portal vein embolization, artificial neural network, computed tomography

## Abstract

Background: This study aimed to evaluate whether hypertrophy after portal vein embolization (PVE) and maximum liver function capacity (LiMAx) are predictable by an artificial neural network (ANN) model based on computed tomography (CT) texture features. Methods: We report a retrospective analysis on 118 patients undergoing preoperative assessment by CT before and after PVE for subsequent extended liver resection due to a malignant tumor at RWTH Aachen University Hospital. The LiMAx test was carried out in a subgroup of 55 patients prior to PVE. Associations between CT texture features and hypertrophy as well as liver function were assessed by a multilayer perceptron ANN model. Results: Liver volumetry showed a median hypertrophy degree of 33.9% (16.5–60.4%) after PVE. Non-response, defined as a hypertrophy grade lower than 25%, was found in 36.5% (43/118) of the cases. The ANN prediction of the hypertrophy response showed a sensitivity of 95.8%, specificity of 44.4% and overall prediction accuracy of 74.6% (*p* < 0.001). The observed median LiMAx was 327 (248–433) μg/kg/h and was strongly correlated with the predicted LiMAx (R^2^ = 0.89). Conclusion: Our study shows that an ANN model based on CT texture features is able to predict the maximum liver function capacity and may be useful to assess potential hypertrophy after performing PVE.

## 1. Introduction

Surgical resection is an important pillar of curative therapy of malignant primary and secondary liver tumors. Even extended liver resections are nowadays possible with reasonable morbidity and mortality rates lower than 30% and 3%, respectively. Functional recovery of the liver remnant is mainly influenced by the future liver remnant (FLR) and the preoperative liver function [1]. Liver volumetry and function tests are routinely used to estimate the future liver remnant volume (FLRV), and volume thresholds exist for a prospectively safe hepatectomy [2]. Patients with insufficient FLRV and/or future liver remnant function (FLRF) must be considered for preoperative hypertrophy induction techniques such as portal vein embolization (PVE) or associating liver partition and portal vein ligation for staged hepatectomy (ALPPS) [3,4]. However, a clinical dropout rate of 20–40% due to slow liver hypertrophy and concurrent tumor progression during the waiting period has been reported [5].

Computed tomography (CT) is a mandatory element of the preoperative workup prior to liver resection in malignant liver disease. It is not only recognized as a sensitive diagnostic tool but has already been shown to be suitable for the characterization of liver functionality [6]. To further develop the value of image-based functional diagnostics, studies have been published that have investigated the relationship between radiomic texture analysis and biological and clinical characteristics using artificial neural networks [7,8]. However, the potential correlation between radiomic texture features and hypertrophy after PVE has not been investigated yet. An estimation prior to PVE, would be of considerable clinical value and change clinical management in patients who are less likely to show significant hypertrophy. Therefore, the aim of this pilot study was to investigate the possibility to predict the hypertrophy potential after PVE using radiomic feature analysis by an artificial neural network. In addition, a subgroup with available dynamic liver function tests was used to investigate the correlation between radiomic features and the enzymatic liver function determined by the LiMAx (maximum liver function capacity) test [9].

## 2. Patients and Methods

### 2.1. Study Population

We report a retrospective analysis on patients undergoing preoperative assessment by CT before and after portal vein embolization for subsequent extended liver resection due to a malignant tumor at RWTH Aachen University Hospital. We included 118 patients who were eligible for PVE between August 2011 and November 2016. Exclusion criteria were unavailability of CT, history of liver surgery or interventions and missing clinical data. Data and imaging of portal vein embolization were prospectively collected, pseudonymized and saved in an institutional database. Radiological imaging was performed 1–9 days prior to PVE and 13–24 days after PVE. The institutional review board approval was obtained before analysis of the data (No. 363/19). In addition, 55 patients underwent a subgroup analysis evaluating the results of the dynamic liver function test LiMAx.

### 2.2. CT Imaging and Volumetry

CT imaging was acquired with a dual-source CT scanner (Siemens Somatom Force, Siemens AG, München, Germany) using the following parameters: 120 kVp tube voltage; 0.5 s gantry rotation; and 5 mm reconstruction thickness. A senior HPB fellow conducted a volumetric analysis using the IntelliSpace Portal 8.0 software tool (Philips healthcare, Amsterdam, The Netherlands). Total liver volume (TLV), tumor volume (TV) and FLR were subsequently computed by the program after manual delineation of margins in every slide. In each of these calculations, TV was considered to be non-functional. The calculated FLR (cFLR) was then computed as described before [10]. Hypertrophy was defined as a proportional increase in cFLR. Patients displaying a hypertrophy of less than 25% were defined as non-responders.

### 2.3. Image Postprocessing

**Figure 1:**
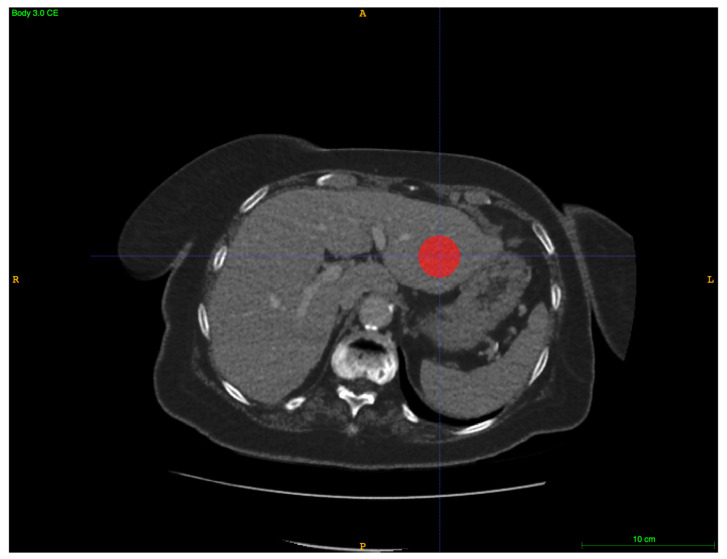
Spherical region of interest (ROIs) in portal venous healthy liver tissue (axial plane) using ITK-snap.

We collected the obtained CT images in the portal venous phase and used ITK-SNAP 3.6.0 (GNU, General Public License, Penn Image Computing and Science Laboratory (PICSL) at the University of Pennsylvania, Philadelphia, PA, USA) to create spherical 3D regions of interest (ROIs) in portal venous healthy liver tissue using a spherical brushing tool, as shown in Figure 1 [11]. The liver tissue delineation was separately exported to NIFTI format. Radiomics feature calculation was conducted using PyRadiomics 2.1.1. (open source, www.radiomics.io, accessed on 9 November 2018). PyRadiomics is a widely used library written in Python and takes both the original CT scan and the created ROI in order to calculate radiomics features from the image inside the ROI only [12]. The purpose of these radiomics features is to capture and quantify the texture characteristics of the tissue inside the ROI.

### 2.4. PVE

PVE was considered in patients with an FLR of less than 30%. The decision for PVE was made by an experienced HPB surgeon in interdisciplinary consensus with a trained interventional radiologist. PVE was performed by the percutaneous transhepatic ipsilateral technique [13]. After transhepatic CT-guided puncture of the right portal branch, a catheter was implanted into the right portal vein. A combination of n-butyl-cyanoacrylate (Braun, Tuttlingen, Germany) and lipiodol (Guerbet, Roissy, France) was used to embolize the right portal vein branches (V-VIII) at a ratio of 1:2 to 1:3. Via repeated portography, successful embolization with free blood flow to the remaining left liver segments was confirmed.

### 2.5. LiMAx Test

The LiMAx test was carried out in 55 patients prior to PVE with borderline FLR or clinically suspected liver parenchymal defect. The LiMAx test is based on hepatic 13C-methacetin (Euriso-top, Saint-Aubin Cedex, France) metabolism by the cytochrome P450 1A2 system (CYP1A2) and was performed as described before [14]. The regular capacity level for liver function is assumed to be >315 μg/kg/h [1].

### 2.6. Statistical Analysis by an Artificial Neural Network

The primary objective of this study was to identify PVE non-responders (hypertrophy < 25%) prior to PVE, and to predict the hypertrophy potential using radiomic texture analysis. Additionally, a subgroup analysis of the patients with measured LiMax was performed to investigate whether a prediction of liver function using texture analysis is feasible. Categorical data are presented as counts and percentages, while data derived from continuous variables are presented as means and interquartile ranges. Associations between radiomic features and hypertrophy as well as liver function were assessed by a multilayer perceptron (MLP) ANN model with a back-propagation algorithm. Using minimum redundancy maximum relevance (mRMR), we selected a total of 51 texture features for hypertrophy analysis and 53 features for liver function analysis by linear regression analysis to construct the final ANN model. It consisted of 3 layers and included 118 cases for hypertrophy prediction and 55 cases for the prediction of liver function. The data were randomly divided into a training and a test sample. Cross-validation was used to minimize overfitting. Predicted LiMAx and measured LiMAx values were then included into a linear regression analysis, and correlations were analyzed using Pearson’s correlation coefficient. Binary prediction of PVE non-responders (hypertrophy < 25%) was evaluated by means of sensitivity and specificity. Receiver operating characteristic (ROC) analysis was carried out by evaluation of the area under the curve (AUC). The level of significance was set to *p* < 0.05, and *p*-values are provided for two-sided testing. Analyses were performed using SPSS Statistics 25 (IBM Corp., Armonk, NY, USA) and MATLAB (MATLAB 2018a, The MathWorks, Inc., Natick, MS, USA).

## 3. Results

**Figure 2:**
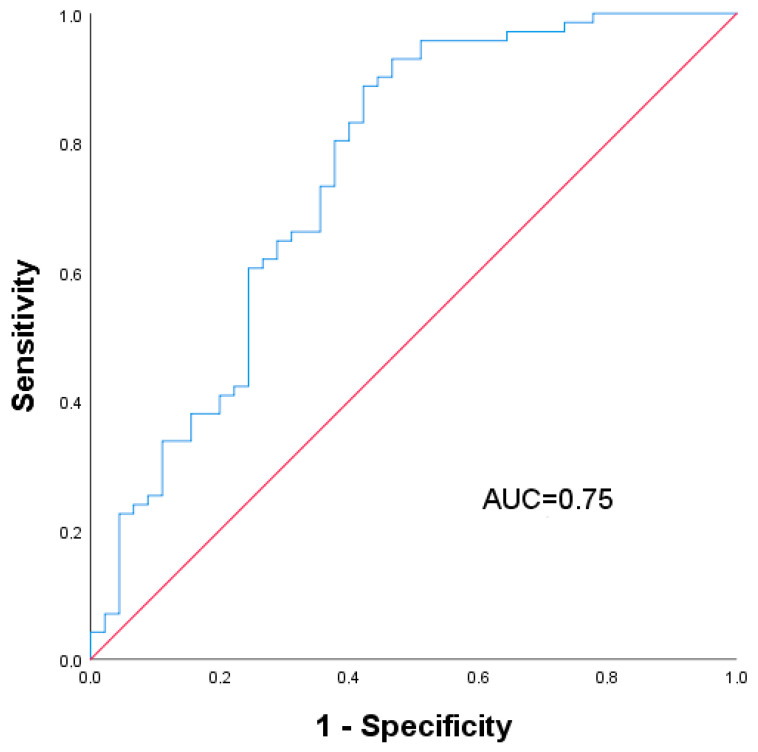
ROC curve analysis of the ANN model predicting hypertrophy > 25% after PVE.

We here analyzed a cohort of 118 patients who underwent PVE and perioperative workup at RWTH Aachen University Hospital between April 2010 and March 2017. Clinical and peri-interventional characteristics are shown in Table 1. A total of 88 male and 30 female patients, with a median age of 65 (56–72) years and a median BMI of 24.9 (22.5–27.7) kg/m^2^, were included in the analysis. The most frequent diagnosis was cholangiocellular carcinoma (CCA) (42.4%), followed by colorectal liver metastasis (CRLM) (39.0%), non-colorectal liver metastasis (LM) (13.6%) and hepatocellular carcinoma (HCC) (5.1%). Chemotherapy was carried out in 31.4% of the cases. In the postoperative histological analysis of the liver, 15.3% of the patients displayed fibrosis, 5.9% steatosis and 5.5% cirrhosis. Liver volumetry showed a median cFLR of 22.9% (17.6–29.3%) prior to PVE and a cFLR of 31.5% (24.0–37.3%) after PVE, with a median hypertrophy degree of 33.9% (16.5–60.4%). Non-response after PVE, defined as a hypertrophy grade lower than 25%, was found in 36.5% (43/118) of the cases. The final three-layer ANN model for the hypertrophy response prediction was then developed and trained with all 118 patients and the extracted radiomic texture features of the CT. The data were randomly divided into a training sample (83 cases, 73%) and a test sample (30 cases, 26.5%). We adjusted our ratio to the limited sample size and chose the ratio as already published [15,16]. The ANN prediction of the hypertrophy response showed a sensitivity of 95.8% and a specificity of 44.4%, with an overall prediction accuracy of 74.6% (*p* < 0.001). The AUC of the ROC curve analysis was 0.75, as shown in Figure 2.

**Figure 3:**
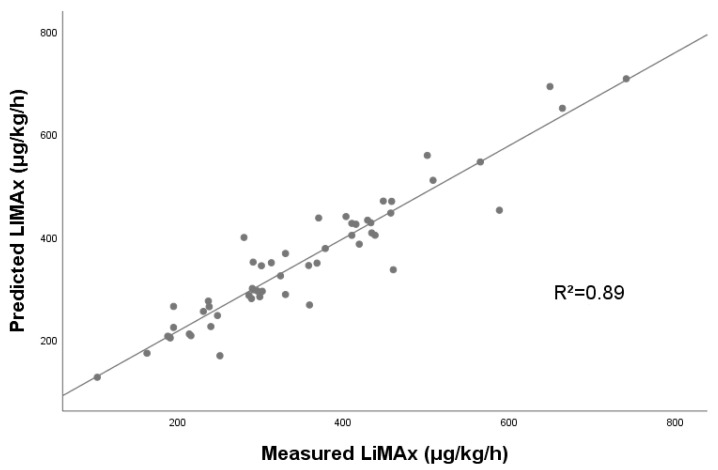
Regression analysis of predicted LiMAx vs. measured LiMAx.

A further subanalysis of 55 cases with available LiMAx data was performed to investigate the prediction of LiMAx by the ANN. Clinical and peri-interventional characteristics are shown in Table 2. Liver steatosis was observed in 5.5% of the cases of this subset, while fibrosis occurred in 10.9% and cirrhosis in 5.5%. Median LiMAx was 327 (248–433) μg/kg/h before performing any intervention or resection. A three-layer ANN with the extracted radiomic texture features was then constructed and trained to predict the maximum liver function capacity. The data were randomly divided into a training sample (41 cases, 75.9%) and a test sample (13 cases, 24.1%). Predicted vs. observed LiMAx values are shown in Figure 3. Linear regression analysis revealed a strong correlation between predicted and observed values, with an R^2^ of 0.89. A score test using Spearman correlation showed a coefficient of 0.723 (CI: 0.536–0.924) and confirmed the observed strong correlation.

## 4. Discussion

In this study, we investigated the predictability of hypertrophy after PVE and LiMax by an ANN model of CT texture features. We demonstrated that an ANN model predicting hypertrophy after PVE based on CT texture features classified the patients, with an accuracy of 74.6%, correctly as either responders or non-responders. In addition, another ANN model based on a subgroup was able to predict LiMAx. The present study is the first to investigate the relationship between CT texture features and PVE-induced hypertrophy of FLR as well as LiMAx by an ANN model. CT has already been described as an accurate tool to assess morphological changes in liver parenchyma such as hepatic fibrosis [17]. However, the role of CT texture analysis in predicting the actual liver function or regeneration potential after PVE remains unclear. A study by Theilig et al. demonstrated the role of gadoxetic acid-enhanced magnetic resonance imaging (MRI) as an imaging-based liver function test before and after PVE to predict post-hepatectomy liver failure [18]. Additionally, studies by Denbo et al. and Schulze-Hagen et al. revealed an association between sarcopenia and poor hypertrophy after PVE which may be caused by liver parenchyma dystrophy [19,20]. To validate the hypothesis concerning whether the hypertrophic potential is predictable using implicit CT imaging features, these were extracted from pre-PVE imaging and transferred into an ANN model. Recent studies already demonstrated the value of ANNs in medical imaging for automated detection and classification of breast masses [21,22]. Finally, our ANN model was able to predict a sufficient post-PVE liver hypertrophy of more than 25%, with acceptable accuracy of 74.6%, based on the portal venous phase of the CT. However, the specificity of 44.4% is certainly limited, meaning that this model in its current configuration is not sufficient for clinical use. Thüring et al. already showed that ANNs allow making a CT-based statement about the parenchyma quality and functional liver status. The authors were able to assess the Child–Pugh class based on multiphase liver CT by a convolutional neural network algorithm [7]. Another hypothesis resulting from this observation was whether the actual liver function determined by LiMAx is predictable by ANNs using CT imaging. Therefore, we further analyzed a subgroup of 55 patients with available LiMAx data. The LiMAx test evaluates the hepatic 13C-methacetin metabolism and has already been used to determine liver function after liver resection and liver transplantation, as well as in liver cirrhosis and non-alcoholic steatohepatitis [1,23,24,25]. While the value of gadoxetic acid-enhanced MRI is widely recognized as an image-based liver function test, data on liver function evaluation by CT using ANNs are very limited [26,27,28]. After constructing the ANN model in our study, a strong correlation between measured and predicted LiMAx values could be demonstrated. Interestingly, our model showed reasonable accuracy in estimating LiMAx based on CT features considering the small number of cases which were analyzed. To the best of our knowledge, this is the first investigation of the prediction of liver function by CT using an ANN model. The construction of an algorithm with high validity could considerably simplify liver function testing, as the current measurement using LiMAx is cost-intensive and requires an invasive measurement on the patient using a breathing mask [9].

Our study has several limitations that need to be discussed. First, this is a retrospective study with a relatively small sample size, which limits its validity, and that may be subject to selection bias. Another limitation is the potential variability of radiomic features when using alternate CT protocols, which may impair the reproducibility [29]. Furthermore, although we performed cross-validation and filtered irrelevant input variables by linear regression analysis to avoid overfitting, the performance and reproducibility of the results of the ANN model need to be tested and validated with a larger cohort in a prospective manner. 

In conclusion, this pilot study shows that an ANN model based on CT texture features is able to predict the maximum liver function capacity and may be useful to assess potential hypertrophy after performing PVE.

## Figures and Tables

**Table 1:** Clinical characteristics pre- and post-PVE.

**Demographics (*n* = 118)**	**#/%**
Gender, m/f (%)	88 (74.6)/30 (25.4)
Age (years)	65 (56–72)
BMI (kg/m^2^)	24.9 (22.5–27.7)
Diagnosis, *n* (%)	
CRLM	46 (39.0)
HCC	6 (5.1)
CCA	50 (42.4)
Other LM	16 (13.6)
ASA, n (%)	
I	23 (19.5)
II	38 (32.2)
III	48 (40.7)
IV	2 (1.7)
**Clinical Characteristics**	**#/%**
Pre-interventional Chemotherapy, *n* (%)	37 (31.4)
Steatosis, *n* (%)	7 (5.9)
Fibrosis, *n* (%)	18 (15.3)
Cirrhosis, *n* (%)	5 (5.5)
**Volumetric Data**	**#/%**
Pre-PVE	
TLV (mL)	1840 (1503–2212)
FLR (mL)	419 (305–554)
cFLR (%)	22.9 (17.6–29.3)
Post-PVE	
TLV (mL)	1824 (1569–2139)
FLR (mL)	534 (436–705)
cFLR (%)	31.5 (24.0–37.3)
Degree of hypertrophy (%)	33.9 (16.5–60.4)

Data presented as median and interquartile range if not noted otherwise. BMI, body mass index; ASA, American Society of Anesthesiologists classification; cFLR, calculated future liver remnant; FLR, future liver remnant; PVE, portal vein embolization; TLV, total liver volume.

**Table 2:** Clinical characteristics of the LiMAx subgroup.

**Demographics (*n* = 55)**	**#/%**
Gender, m/f (%)	39 (70.9)/16 (29.1)
Age (years)	64 (53–69)
BMI (kg/m^2^)	24.8 (21.9–27.7)
Diagnosis, *n* (%)	
CRLM	23 (41.8)
HCC	3 (5.5)
CCA	19 (34.5)
Other LM	10 (18.2)
ASA, *n* (%)	
I	15 (27.3)
II	15 (27.3)
III	19 (34.5)
IV	0 (0)
**Clinical Characteristics**	**#/%**
Pre-interventional Chemotherapy	17 (30.9)
Steatosis, *n* (%)	3 (5.5)
Fibrosis, *n* (%)	6 (10.9)
Cirrhosis, *n* (%)	3 (5.5)
**Liver Function**	**#**
LiMAx (µg/kg/h)	327 (248–433)

Data presented as median and interquartile range if not noted otherwise. BMI, body mass index; ASA, American Society of Anesthesiologists classification.

## Data Availability

The datasets generated and analyzed during the current study are not publicly available due to the local privacy policy on clinical data but are available from the corresponding author on reasonable request.

## Abbreviations

ALPPS	Associating liver partition with portal vein ligation for staged hepatectomy
ASA	American Society of Anesthesiologists
BMI	Body mass index
CCA	Cholangiocellular carcinoma
CRLM	Colorectal liver metastases
CT	Computed tomography
FLR	Future liver remnant
FLRF	Future liver remnant function
FLRV	Future liver remnant volume
HCC	Hepatocellular carcinoma
INR	International normalized ratio
LiMAx	Maximum liver function capacity
LM	Non-colorectal liver metastasis
MLP	Multilayer perceptron
MRI	Gadolinium-based magnetic resonance imaging
PVE	Portal vein embolization
ROI	Region of interest

## References

[1] Lock J.F., Malinowski M., Seehofer D., Hoppe S., Röhl R.I., Niehues S.M., Neuhaus P., Stockmann M. (2012). Function and volume recovery after partial hepatectomy: Influence of preoperative liver function, residual liver volume, and obesity. Langenbeck’s Arch. Surg. Dtsch. Ges. Fur Chir..

[2] Khan A.S., Garcia-Aroz S., Ansari M.A., Atiq S.M., Senter-Zapata M., Fowler K., Doyle M.B., Chapman W.C. (2018). Assessment and optimization of liver volume before major hepatic resection: Current guidelines and a narrative review. Int. J. Surg..

[3] Tschuor C., Croome K.P., Sergeant G., Cano V., Schadde E., Ardiles V., Slankamenac K., Clariá R.S., de Santibaňes E., Hernandez-Alejandro R. (2013). Salvage parenchymal liver transection for patients with insufficient volume increase after portal vein occlusion—An extension of the ALPPS approach. Eur. J. Surg. Oncol..

[4] Shindoh J., Vauthey J.N., Zimmitti G., Curley S.A., Huang S.Y., Mahvash A., Gupta S., Wallace M.J., Aloia T.A. (2013). Analysis of the efficacy of portal vein embolization for patients with extensive liver malignancy and very low future liver remnant volume, including a comparison with the associating liver partition with portal vein ligation for staged hepatectomy approach. J. Am. Coll. Surg..

[5] Shindoh J., Tzeng C.W., Aloia T.A., Curley S.A., Huang S.Y., Mahvash A., Gupta S., Wallace M.J., Vauthey J.N. (2014). Safety and efficacy of portal vein embolization before planned major or extended hepatectomy: An institutional experience of 358 patients. J. Gastrointest. Surg..

[6] Yeom S.K., Lee C.H., Cha S.H., Park C.M. (2015). Prediction of liver cirrhosis, using diagnostic imaging tools. World J. Hepatol..

[7] Thüring J., Rippel O., Haarburger C., Merhof D., Schad P., Bruners P., Kuhl C.K., Truhn D. (2020). Multiphase CT-based prediction of Child-Pugh classification: A machine learning approach. Eur. Radiol. Exp..

[8] House M.J., Bangma S.J., Thomas M., Gan E.K., Ayonrinde O.T., Adams L.A., Olynyk J.K., St Pierre T.G. (2015). Texture-based classification of liver fibrosis using MRI. J. Magn. Reson. Imaging JMRI.

[9] Stockmann M., Lock J.F., Riecke B., Heyne K., Martus P., Fricke M., Lehmann S., Niehues S.M., Schwabe M., Lemke A.J. (2009). Prediction of postoperative outcome after hepatectomy with a new bedside test for maximal liver function capacity. Ann. Surg..

[10] Bednarsch J., Czigany Z., Sharmeen S., van der Kroft G., Strnad P., Ulmer T.F., Isfort P., Bruners P., Lurje G., Neumann U.P. (2020). ALPPS versus two-stage hepatectomy for colorectal liver metastases—A comparative retrospective cohort study. World J. Surg. Oncol..

[11] Yushkevich P.A., Piven J., Hazlett H.C., Smith R.G., Ho S., Gee J.C., Gerig G. (2006). User-guided 3D active contour segmentation of anatomical structures: Significantly improved efficiency and reliability. NeuroImage.

[12] Van Griethuysen J.J.M., Fedorov A., Parmar C., Hosny A., Aucoin N., Narayan V., Beets-Tan R.G.H., Fillion-Robin J.C., Pieper S., Aerts H. (2017). Computational Radiomics System to Decode the Radiographic Phenotype. Cancer Res..

[13] Adam R., Laurent A., Azoulay D., Castaing D., Bismuth H. (2000). Two-stage hepatectomy: A planned strategy to treat irresectable liver tumors. Ann. Surg..

[14] Alizai P.H., Haelsig A., Bruners P., Ulmer F., Klink C.D., Dejong C.H.C., Neumann U.P., Schmeding M. (2018). Impact of liver volume and liver function on posthepatectomy liver failure after portal vein embolization- A multivariable cohort analysis. Ann. Med. Surg..

[15] Monti C.B., Codari M., van Assen M., De Cecco C.N., Vliegenthart R. (2020). Machine Learning and Deep Neural Networks Applications in Computed Tomography for Coronary Artery Disease and Myocardial Perfusion. J. Thorac. Imaging.

[16] Ding N., Hao Y., Wang Z., Xuan X., Kong L., Xue H., Jin Z. (2020). CT texture analysis predicts abdominal aortic aneurysm post-endovascular aortic aneurysm repair progression. Sci. Rep..

[17] Yoon J.H., Lee J.M., Klotz E., Jeon J.H., Lee K.B., Han J.K., Choi B.I. (2015). Estimation of hepatic extracellular volume fraction using multiphasic liver computed tomography for hepatic fibrosis grading. Investig. Radiol..

[18] Theilig D., Steffen I., Malinowski M., Stockmann M., Seehofer D., Pratschke J., Hamm B., Denecke T., Geisel D. (2019). Predicting liver failure after extended right hepatectomy following right portal vein embolization with gadoxetic acid-enhanced MRI. Eur. Radiol..

[19] Denbo J.W., Kim B.J., Vauthey J.N., Tzeng C.W., Ma J., Huang S.Y., Chun Y.S., Katz M.H.G., Aloia T.A. (2020). Overall Body Composition and Sarcopenia Are Associated with Poor Liver Hypertrophy Following Portal Vein Embolization. J. Gastrointest. Surg..

[20] Schulze-Hagen M., Truhn D., Duong F., Keil S., Pedersoli F., Kuhl C.K., Lurje G., Neumann U., Isfort P., Bruners P. (2020). Correlation between Sarcopenia and Growth Rate of the Future Liver Remnant after Portal Vein Embolization in Patients with Colorectal Liver Metastases. Cardiovasc. Interv. Radiol..

[21] Rouhi R., Jafari M., Kasaei S., Keshavarzian P. (2015). Benign and malignant breast tumors classification based on region growing and CNN segmentation. Expert Syst. Appl..

[22] Dhungel N., Carneiro G., Bradley A.P. Automated mass detection in mammograms using cascaded deep learning and random forests. Proceedings of the 2015 International Conference on Digital Image Computing: Techniques and Applications (DICTA).

[23] Malinowski M., Jara M., Lüttgert K., Orr J., Lock J.F., Schott E., Stockmann M. (2014). Enzymatic liver function capacity correlates with disease severity of patients with liver cirrhosis: A study with the LiMAx test. Dig. Dis. Sci..

[24] Stockmann M., Lock J.F., Malinowski M., Seehofer D., Puhl G., Pratschke J., Neuhaus P. (2010). How to define initial poor graft function after liver transplantation—A new functional definition by the LiMAx test. Transpl. Int..

[25] Alizai P.H., Wendl J., Roeth A.A., Klink C.D., Luedde T., Steinhoff I., Neumann U.P., Schmeding M., Ulmer F. (2015). Functional Liver Recovery after Bariatric Surgery—A Prospective Cohort Study with the LiMAx Test. Obes. Surg..

[26] Verloh N., Haimerl M., Zeman F., Schlabeck M., Barreiros A., Loss M., Schreyer A.G., Stroszczynski C., Fellner C., Wiggermann P. (2014). Assessing liver function by liver enhancement during the hepatobiliary phase with Gd-EOB-DTPA-enhanced MRI at 3 Tesla. Eur. Radiol..

[27] Haimerl M., Verloh N., Fellner C., Zeman F., Teufel A., Fichtner-Feigl S., Schreyer A.G., Stroszczynski C., Wiggermann P. (2014). MRI-based estimation of liver function: Gd-EOB-DTPA-enhanced T1 relaxometry of 3T vs. the MELD score. Sci. Rep..

[28] Yamada A., Hara T., Li F., Fujinaga Y., Ueda K., Kadoya M., Doi K. (2011). Quantitative evaluation of liver function with use of gadoxetate disodium-enhanced MR imaging. Radiology.

[29] Berenguer R., Pastor-Juan M.D.R., Canales-Vazquez J., Castro-Garcia M., Villas M.V., Mansilla Legorburo F., Sabater S. (2018). Radiomics of CT Features May Be Nonreproducible and Redundant: Influence of CT Acquisition Parameters. Radiology.

